# The roles of Eph receptors, neuropilin-1, P2X7, and CD147 in COVID-19-associated neurodegenerative diseases: inflammasome and JaK inhibitors as potential promising therapies

**DOI:** 10.1186/s11658-022-00311-1

**Published:** 2022-02-02

**Authors:** Hamidreza Zalpoor, Abdullatif Akbari, Azam Samei, Razieh Forghaniesfidvajani, Monireh Kamali, Azadeh Afzalnia, Shirin Manshouri, Fatemeh Heidari, Majid Pornour, Majid Khoshmirsafa, Hossein Aazami, Farhad Seif

**Affiliations:** 1 American Association of Kidney Patients, Tampa, FL USA; 2 Neuroscience Research Center, Iran University of Medical Sciences, Tehran, Iran; 3 Shiraz Neuroscience Research Center, Shiraz University of Medical Sciences, Shiraz, Iran; 4 Network of Immunity in Infection, Malignancy and Autoimmunity (NIIMA), Universal Scientific Education and Research Network (USERN), Tehran, Iran; 5 Department of Laboratory Sciences, School of Paramedical Sciences, Kashan University of Medical Sciences, Kashan, Iran; 6 Rajaei Cardiovascular Medical and Research Center, Iran University of Medical Sciences, Tehran, Iran; 7 Immunology Department, Faculty of Medicine, Tarbiat Modares University, Tehran, Iran; 8 Department of Oncology, School of Medicine, University of Maryland, Maryland, USA; 9 Department of Immunology, School of Medicine, Iran University of Medical Sciences, Iran, Iran; 10 Endocrinology and Metabolism Research Center, Endocrinology and Metabolism Clinical Sciences Institute, Tehran University of Medical Sciences, Tehran, Iran; 11 Department of Immunology and Allergy, Academic Center for Education, Culture, and Research (ACECR), Enghelab St., Aboureyhan St., Vahid Nazari Crossroad, P17, 1315795613 Tehran, Iran

**Keywords:** COVID-19, CNS, Ephrin, Neuropilin-1, P2X7, CD147, Cytokine, Jak, Inflammasome, Neurodegenerative diseases, Alzheimer’s disease, Parkinson’s disease

## Abstract

The novel coronavirus disease 2019 (COVID-19) pandemic has spread worldwide, and finding a safe therapeutic strategy and effective vaccine is critical to overcoming severe acute respiratory syndrome coronavirus 2 (SARS-CoV-2). Therefore, elucidation of pathogenesis mechanisms, especially entry routes of SARS-CoV-2 may help propose antiviral drugs and novel vaccines. Several receptors have been demonstrated for the interaction of spike (S) protein of SARS-CoV-2 with host cells, including angiotensin-converting enzyme (ACE2), ephrin ligands and Eph receptors, neuropilin 1 (NRP-1), P2X7, and CD147. The expression of these entry receptors in the central nervous system (CNS) may make the CNS prone to SARS-CoV-2 invasion, leading to neurodegenerative diseases. The present review provides potential pathological mechanisms of SARS-CoV-2 infection in the CNS, including entry receptors and cytokines involved in neuroinflammatory conditions. Moreover, it explains several neurodegenerative disorders associated with COVID-19. Finally, we suggest inflammasome and JaK inhibitors as potential therapeutic strategies for neurodegenerative diseases.

## Introduction

Severe acute respiratory syndrome coronavirus 2 (SARS-CoV-2) was initially reported in Wuhan, Hubei Province, China, on 31 December 2019. SARS-CoV-2 causes novel coronavirus disease 2019 (COVID-19) [1, 2]. SARS-CoV-2 has spread much faster than SARS because of the high affinity of SARS-CoV-2 spike proteins to angiotensin-converting enzyme (ACE2) and its viral load [3]. Several human respiratory viruses are neuroinvasive and neurotropic, with potential neuropathological consequences in vulnerable populations. Unraveling the underlying mechanisms of neuroinvasion due to respiratory viruses, especially SARS-CoV-2, is essential to evaluate potentially pathological short- and long-term consequences.

Several human respiratory viruses, including influenza virus, SARS-CoV-1, and SARS-CoV-2 are neurotropic and neuroinvasive; therefore, they can naturally reach the central nervous system (CNS). Three routes have been described for the CNS being affected by COVID-19 infection: (1) direct viral infection of nervous tissue or viral injuries resulting from systemic illnesses; (2) injury occurring when the immune system overreacts, for example in a cytokine storm [4, 5] such as in Alzheimer’s disease (AD), hemorrhagic strokes, and Guillain–Barré syndrome (GBS). It is possible for cytokines to pass through the blood–brain barrier (BBB) and cause acute necrotizing encephalopathy; and (3) unintended responses of the host’s immune response following acute infection, for example peripheral nervous system (PNS) injury in GBS.

These respiratory viruses can potentially associate with neurological symptoms such as GBS, increasing the risk of neurodegenerative disease such as AD, Parkinson’s disease (PD), ischemic stroke, and multiple sclerosis (MS), as well as neuropsychiatric symptoms such as mania, anxiety, depression, psychosis, delirium, and insomnia [6, 7]. In this review, we describe potentially pathological mechanisms of SARS-CoV-2 infection in the CNS, including entry receptors and cytokines involved in neuroinflammatory conditions. In the following, we report some neurodegenerative disorders as a result of COVID-19, as well as novel markers and therapeutic strategies that may help diagnose and manage these disorders by possibly targeting these molecules.

### How does SARS-CoV-2 reach the CNS?

SARS-CoV-2 may use three main ways to reach the CNS, as follows: (1) the virus can use olfactory bulb neurons (or other nerve tracts) to travel from the periphery into the brain, (2) the virus may also penetrate the BBB to gain access to the brain [8, 9], and (3) through neuronal pathways [10]. Some studies have demonstrated that patients infected with SARS-CoV-2 are possibly faced with multiple organ systems infection [11]. ACE2 is known as the specific SARS-CoV-2 receptor expressed in the human brain. Other potential receptors on the surface of host cells include Eph receptors and ephrin ligands [12], neuropilin-1 (NRP-1) [13, 14], transmembrane serine protease 2 (TMPRSS2) [15], P2X7 [16], and CD147 [17]. Symptoms associated with the pituitary gland include headaches, impaired pituitary function, and vision changes. Cerebellum and cortex damage can cause neurological symptoms such as ataxia, dizziness, and impaired consciousness. Thus one reason for patients’ cognitive and neurological impairment may be the susceptibility of these brain regions to SARS-CoV-2. Given that SARS-CoV-2 infection may result in different pathological changes and symptoms depending on the type of cell and region of the brain, the following novel markers and clinical findings may provide some insight into the mechanisms involved in COVID-19-associated neurodegenerative diseases [15].

### ACE2

Angiotensin-converting enzyme (ACE), a dicarboxypeptidase vasoconstrictor, can critically regulate the blood pressure [18]. Angiotensin-converting enzyme (ACE2) is known as the specific SARS-CoV-2 receptor, which is also expressed in the human brain [3]. SARS-CoV-2 coat contains the spike protein, which has a receptor-binding region that directly binds to the ACE2 extracellular domain. Studies have revealed that the affinity of the SARS-CoV-2 spike protein for human ACE2 is higher than the spike protein of SARS-CoV [19], and new analysis indicates that the newly emerged variant Omicron RBD binds more strongly to human ACE2 than the original strain [20]. In COVID-19, ACE2 plays a crucial role. It is not only a specific receptor for viral entry, but is also involved in postinfection processes, including stimulation of intracellular signaling pathways [3], immune system response, cytokine secretion, and replication of the viral genome [21]. It seems that ACE2 may have a critical role in the target organ of the SARS-CoV-2 infection because SARS-CoV-2 binds to the ACE2 receptor before entering the host cell. ACE2 is detectable in some CNS cell types, including neurons, microglia, astrocytes, and oligodendrocytes [22]. Microglial cells are bone marrow-derived macrophage-like cells that are resident within the CNS. Microglial cells exert their roles in mediating neuronal immune interactions under both pathological and physiological conditions [23]. Upon microglia activation, they secrete chemokines, improve BBB permeability, and cause an inflammatory process in the CNS. They have a role in cytokine storm formation by secretion of tumor necrosis factor-alpha (TNF-α), interleukin-1 (IL-1), and IL-6 [24]. Astrocytes are endogenous cells of CNS that play critical roles in immune responses [25]. Genes associated with inflammation and astrogliosis (such as* IFITM3* and* GFAP*) and the secreted neurotoxic factor chitinase 3-like 1 (CHI3L1) are upregulated in association with COVID-19. Studies have shown that patients with severe COVID-19 have significant dysregulation of genes involved in neurotransmission and synaptic organization-related astrocyte cluster [26].

## Ephrin ligands and Eph receptors

In humans, the largest superfamily of receptor tyrosine kinases (RTKs) is the erythropoietin-producing hepatocellular (Eph) receptor. A wide variety of cellular processes such as adhesion, proliferation, and differentiation to cell migration are controlled by the RTK pathway known as ephrin–Eph. It is also noteworthy that the Eph receptors and their ligands, Eph family receptor-interacting protein (ephrin), are broadly expressed and highly conserved by a diversity of cells in a wide range of organisms [27]. Activation of Eph receptors takes place by binding their ligands, ephrin. The ligand-binding region is the extracellular domain of Eph receptors, while the intracellular domain contains regulatory regions and the protein kinase domain. As a result of their activation by their ligands, the tyrosine residues of receptors are phosphorylated, acting as the assembly and activation site of intracellular signaling proteins (or adapters). Eph receptors are classified into two classes based on their binding preferences: EphA and EphB. EphA receptors bind to ephrin A ligands, while EphB receptors bind to ephrin B ligands. EphA receptors consist of ten members, called EphA1–EphA10, while EphB receptors consist of six members, defined as EphB1–EphB6 [27]. Several RNA viruses have been shown to utilize EphA2 as their main entry receptor into host cells [28]. Furthermore, the Eph receptor and ephrin ligand has been found to be a cell signaling pathway protein that may exert its role as an alternative coreceptor for viral entry or modulation of signaling cascades during SARS-CoV-2, SARS-CoV, and Middle East respiratory syndrome (MERS) infections [12].

The Eph/ephrin family is found on the surface of a number of different cells, and these proteins have important roles in injury (particularly wound healing and ischemia–reperfusion injury) and inflammation [27]. Eph and ephrin are widely expressed in the CNS, and their pathological role in neuropsychiatric, neurodevelopmental, and neurodegenerative diseases is well established. Several types of CNS cells have been shown to express Eph and ephrin, including hippocampal astrocytes, subgranular zone (SGZ) of the hippocampal dentate gyrus, type 2a in neural stem cells (NSCs), type 3 in neuroblasts, type 2b in neuronal precursors [29], and type 3a in astrocytes [30]. It has also been shown that reactive astrocytes in MS active plaques express ephrin A3, Eph A1, and Eph A4 [31]. Ephrins and Eph receptors are typically expressed in neurons; however, some ephrins may also be expressed in glial cells [32].

In the following, we explain the ways by which SARS-CoV-2 influences these cell surface proteins. Several viruses have been found to be able to enter cells via membrane fusion and endocytosis using Ephs and ephrins as entry receptors. In addition, Eph and ephrin proteins are also involved in viral transmission via vectors, viral replication, or persistence. For example, neurological damage can be caused by viral infection, such as Nipah virus (NiV), Hendra virus (HeV), and Epstein–Barr virus (EBV) [27].

Ephrin signaling can occur in three directions: forward, reverse, and bidirectional, which distinguishes Ephrin–Eph receptor signaling from other RTK signaling pathways [27]. Upon activation, adapter proteins bind to the receptor and transmit signals further downstream into the cell. Several adapter proteins interact with the Eph receptors such as Ras-GTPase-activating protein (RasGAP), phosphatidylinositol 3-kinase (PI3K), and Janus kinase 2 (JaK2) [27, 33]. The JAK-signal transducer and activator of transcription (STAT) pathway is the major signaling pathway in cytokine-mediated immune system regulation [34]. It has also been demonstrated that astrocytes activated by ephrin-B1 showed an increased level of STAT3 phosphorylation in vitro [35]. It has been reported that TNF-α, which is overexpressed during COVID-19, can induce the expression of ephrin A3 in endothelial cells via the c-Jun N-terminal kinases (JNK) and p38-mitogen-activated protein kinases (MAPK) signaling pathways [36, 37], and in a recent study, it was revealed that the expression of ephrin A1 was significantly increased during SARS-CoV-2 infection [38]. On the other hand, it has recently been shown that ephrin ligand can be a potential receptor for modulation of signaling cascades during SARS-CoV-2 infection [12]. According to these studies, Eph receptor tyrosine kinases and their ligands, ephrins, modulate dendritic spine morphogenesis, synaptic formation, and maturation, as well as synaptic plasticity. As a result, it seems that SARS-CoV-2 may stimulate signaling pathways that lead to the emergence or aggravation of neurodegenerative diseases during COVID-19.

### Neuropilin 1

Neuropilin 1 (NRP-1) is one of two homologous neuropilins that play pivotal roles in physiological and pathological conditions. NRP-1 has two isoforms, including secretory (as truncated or soluble NRP-1) and transmembrane isoforms. The former circulates freely in the body fluid, while the latter interacts with various ligands and has different functions [39].

NRP-1 exerts its role as a receptor for signaling ligands including vascular endothelial growth factor (VEGF, particularly VEGF-A), semaphorins, transforming growth factor-beta (TGF-β), integrins, and plexins [39]. NRP-1 has a significant multisystem role in viral entry, angiogenesis, tumor progression, axonal guidance within the CNS and PNS, and immune function [40]. Diverse expression and functional characteristics of NRP-1 make it an appropriate extracellular target for SARS-CoV-2, and potentially contribute to the multisystem implications of COVID-19. Specifically, the hippocampal formation consistently expressed the highest levels of NRP-1 genes and proteins [39]. Consequently, NRP-1 may serve as an additional SARS-CoV-2 infection mediator involved in the neurologic manifestations of COVID-19 [41]. A study by Davies et al. demonstrated that NRP-1 is expressed in the CNS, affecting olfactory-related regions such as the olfactory tubercles and paraolfactory gyri, indicating the potential role of NRP-1 as another entry route for SARS-CoV-2 in neurologic complications of COVID-19 [13]. Furthermore, the cardiovascular, gastrointestinal, respiratory, and neurological functions of the brainstem are possibly impaired due to brainstem damage even in mild cases of COVID-19, and may lead to long-lasting pathological outcomes [42]. NRP-1 is also expressed by adipose tissue macrophages, retinal vasculature, CD8^+^ T cells, regulatory T cells, and alveolar, bronchial, and vascular macrophages [43]. Furthermore, NRP-1 is frequently expressed in the respiratory and olfactory epithelium, as well as olfactory neurons. Notably, this may be suggested as a direct path for SARS-CoV-2 to penetrate via the olfactory bulb and to traverse into the CNS, thereby disrupting olfaction and resulting in anosmia in the context of COVID-19 [39]. Two other studies, by Daly et al. and Cantuti-Castelvetri et al., identified NRP-1 as another route of SARS-CoV-2 entry [13, 14].

### P2X7 receptor

The P2X7 receptor is a cation channel that activates at high concentrations of adenosine triphosphate (ATP). As a result of long-term P2X7 activation, it forms a wide pore that causes cell death and enhances ATP release into the extracellular environment. The P2X7 receptor is widely expressed in the CNS, including in the hippocampus, frontal cortex, amygdala, and striatum; regions that are associated with neurodegenerative diseases and neuropsychiatric disorders [44]. It is possible that SARS-CoV-2 alters brain function by reaching the CNS or by resulting in cytokine storms. The outcome is a neuroinflammatory process characterized by neuron demyelination, microglia hyperactivation, and astrocyte stimulation. Molecularly, cells in distress release proinflammatory cytokines and ATP. ATP activates P2X7 receptors that are typically expressed in microglia and astrocytes, leading to increased Ca^2+^ influx and glutamate release. In microglia, P2X7 receptor activation mediates K^+^ efflux, which may promote Nod-like receptor family pyrin domain-containing 3 (NLRP3) inflammasome activation [16]. Consequently, the activation of the P2X7 receptor, as well as SARS-CoV-2 invasion via ACE2, Eph or ephrin ligand, NRP-1, CD147, or TMPRSS2, activates the NLRP3 inflammasome in host cells. Therefore, hyperactivation of these pathways triggers caspase 1 activation and mature IL-1β and IL-18 release, which may contribute to a type of cell death named pyroptosis. Further, activation of the P2X7 receptor by ATP stimulates the activation of both MAPKs (p38, ERK, and JNK) and nuclear factor of activated T cells (NFAT) [45]. Activation of the P2X7 receptor stimulates the release of other inflammatory cytokines and chemokines, including TNF-α, IL-6, CCL2, CCL3, and CXCL2 [16, 45, 46].

### CD147

**Fig. 1:**
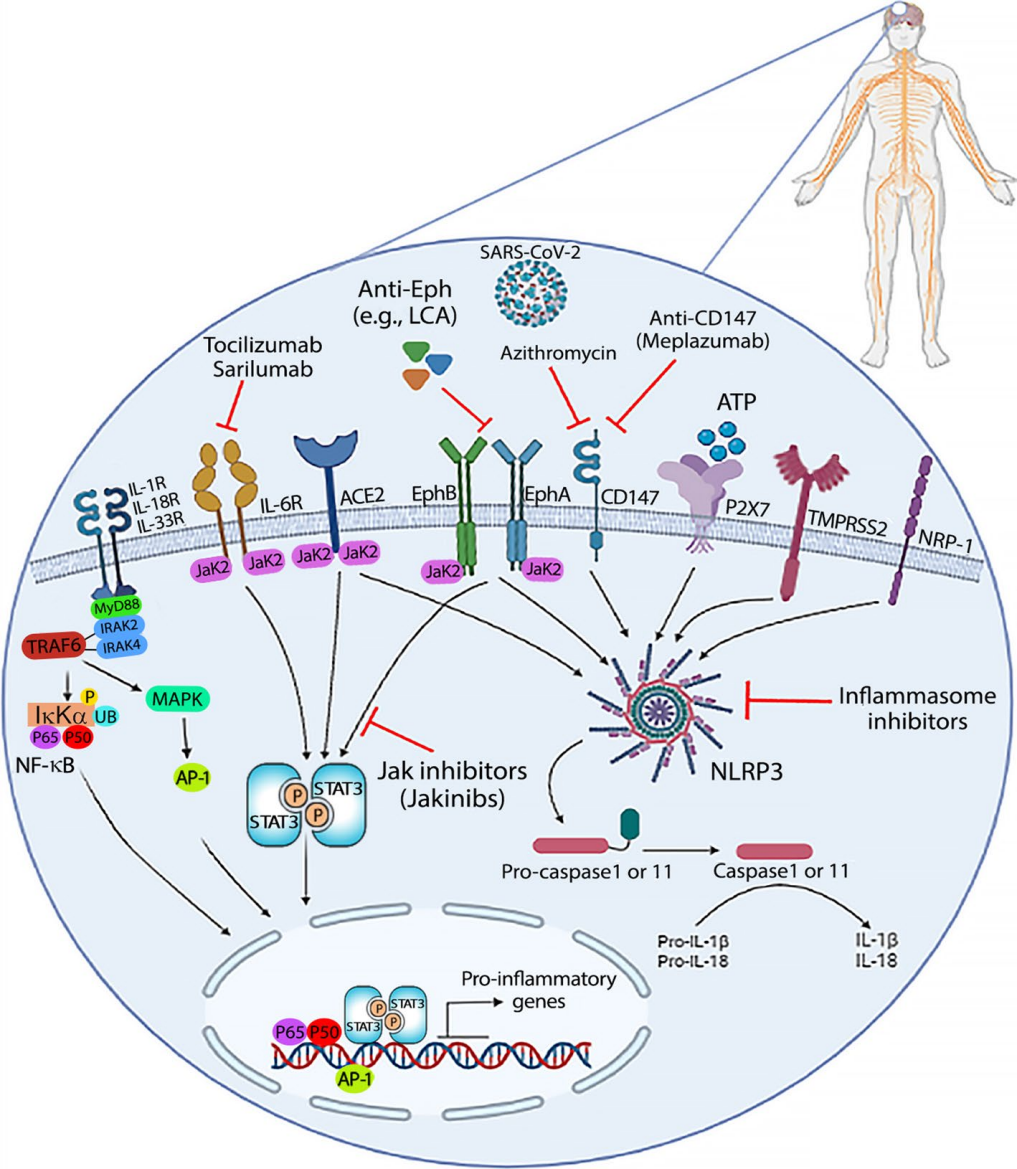
ACE2, Eph receptors and ephrin ligands, neuropilin-1 (NRP-1), transmembrane serine protease 2 (TMPRSS2), P2X7, and CD147 are shown as entry receptors for SARS-CoV-2 in neurodegenerative diseases. ACE2, EphA/B receptors, and IL-6 receptor use JaK2/STAT3 for signal transduction; thus they may be targeted by JaK inhibitors (JaKinibs). Moreover, ACE2, EphA/B receptors, TMPRSS2, (NRP-1), P2X7, and CD147 can activate NLRP3 inflammasome to secrete IL-1β and IL-18; therefore, they may be targeted by inflammasome inhibitors or IL-1β and IL-18 monoclonal antibodies or antagonists

CD147 (HAb18G, also called EMMPRIN) is a transmembrane glycoprotein and a member of the immunoglobulin superfamily [47]. A variety of organs and cells in the body contain CD147, including the lungs, T cells, endothelial cells, and brain [48, 49]. CD147 has been demonstrated to be involved in human immunodeficiency virus (HIV)-1 infection by interacting with virus-associated cyclophilin A, and in other viral infections, especially SARS-CoV-2 infection, it is not directly bound to SARS-CoV-2 [50, 51]. In comparison to ACE2, the cerebral nervous system was more likely to be infected with SARS-CoV-2 through the CD147 receptor and TMPRSS2 protease. Furthermore, given that both CD147 and TMPRSS2 are necessary for SARS-CoV-2 infection, neuron and microglia may be susceptible to SARS-CoV-2 infection; however, astrocytes would be less susceptible. Studies have found that TMPRSS2 and CD147 mRNA levels were higher in pituitary, cerebellum, and cortex of a mouse brain [15] (Fig. 1).

A number of pathways are thought to be involved in CD147 action, including MAPK p38, ERK-1/2, PI3K, and NF-κB [52, 53]. CD147 stimulation appears to trigger ERK phosphorylation, IκB phosphorylation-associated degradation, and nuclear translocation of NF-κB p50 and p65 subunits [54]. In various cells, CD147 mediates the inflammatory activation, including macrophages, that results in the induction of MMP-9 expression, and expression of proinflammatory cytokines and chemokines in endothelial cells [54], which may contribute to the pathogenesis of inflammatory diseases. SARS-CoV-2 entry receptors, such as CD147 and ACE2, are thought to allow the virus to enter the cell cytoplasm and activate the NLRP3 inflammasome, resulting in the cleavage of IL-1β and IL-18 cytokines [55–57].

### Cytokines

In COVID-19 patients, high levels of proinflammatory cytokines such as IL-6, IL-1β, IL-18, IL-33 and TNF-α/β have been reported in blood plasma. During COVID-19, these proinflammatory cytokines produced by different infected cells are spread in general circulation and then pass from the BBB and affect different cell types of CNS by stimulating their receptors on the surface of these cells. Furthermore, direct SARS-CoV-2 infection of some CNS cell types can lead to their activation and the production of proinflammatory cytokines. Interleukin receptors are cell surface receptors in the CNS, which can activate the canonical signaling pathway of NF-κB [58].

*IL-1 and IL-1 receptor (IL-1R)* Microglia are a significant source of the IL-1 superfamily, including IL-1α, IL-1β, IL-6, IL-8, IL-18, and IL-33, and also have prominent roles in neuroinflammation [59, 60]. IL-1 receptor type I (IL-1RI), IL-1RII, and IL-receptor accessory protein (IL-1R-AcP) are three types of IL-1 receptors [61]. IL-1 attach to IL-1R and forms a complex with IL-1R-AcP. Then NF-κB, p38, JNK, extracellular signal‑regulated kinases (ERKs) and MAPK are activated by MyD88, IRAK, and TRAF6 through the TIR domain of IL-1R. This induces various enzymes, such as phospholipase A2, cyclooxygenase 2, and nitric oxide (NO) synthase that stimulate prostaglandin E2 and NO. Fever, neutrophilia, thrombocytosis, and the production of acute‑phase proteins are IL-1 systemic effects. IL-1 also plays a key role in Th17 differentiation [62].

*IL-6 and IL-6R* IL-6 are excreted by microglia, neurons, astrocytes, and peripheral monocytes [63]. IL-6R has two types of molecules for activity: IL-6R (also known as IL-6Rα, gp80, or CD126) and gp130 (also referred to as IL-6Rβ or CD130). IL-6R is divided into membrane-bound IL-6R/gp130 (mIL-6R) (mgp130) and soluble IL-6R/gp130 (sIL-6R) (sgp1130) [64]. IL-6 attaches to IL-6R and forms a complex with mgp130 to induce AKT-PI3K and RAS-RAF pathways in glial cells of the lateral olfactory tract, granular cell layer of the cerebellum, and astrocyte, and if it binds to sgp130, it inhibits signal transduction [64–66]. IL-6 induces fever and adrenocorticotropic hormone (ACTH), as well as being involved in Parkinson’s disease, MS, and AD [67].

*IL-18 and IL-18R* IL-18 is a member of the IL-1 superfamily, which is secreted in its active form following maturation by caspase 1 in response to inflammatory and infectious stimuli. Isoforms of both IL-18Rα and IL-18Rβ were recently described in vivo in the CNS. Canonical IL-18 action occurs via recruitment of MyD88. This then leads to activation of the IRAK/TRAF6 pathway and ultimately NF-κB activation and subsequent modulation of gene transcription [68]. In addition, studies have suggested that MAPK and PI3K have a role in IL-18 signaling [68]. Binding of IL-18 to IL-18R increases NF-κB and causes induction of astrocyte hypertrophy [69]. Evidence shows that IL-18 has a prominent role in neuroinflammation and neurodegenerative disease such as AD under pathological conditions such as viral infections [68, 70]. IL-18 has a key role in activating the function of microglia and astrocytes [71, 72], and activation of microglia leads to production of proinflammatory cytokines such as IL-1β, IL-6, IL-8, IL-18, and IL-33 [59].

*IL-33 and IL-33R* IL-33 is a member of the IL-1 family of cytokines, with the receptors ST2 and IL-1 receptor accessory protein (IL-1RAcp) [73]. In CNS, astrocytes, oligodendrocytes, and epithelial cells produce IL-33 but not microglia or neurons. In fact, it has been demonstrated that microglia and astrocytes express IL-33 receptors (ST2, IL-1RAcp, and sST2), but IL-1RAcp is expressed by neurons only [73, 74]. IL-33 activates Th2 to produce Th2 cytokines and express ST2 [73]. In CNS, microglial cells exert their roles as the principal cells of the innate immune system and also roles in neuroinflammation and neuroprotection. IL-33 induces microglia proliferation and is a microglia-activator cytokine that may play a critical role in neuroinflammatory processes in the CNS [73]. IL-33 activates microglia via its receptors and promotes MAPK and NF-κB signaling pathways; increases production of proinflammatory cytokines, such as IL-1β and TNF-α, by microglia; and enhances phagocytosis of microglia [73, 75].

*TNF-α and TNFRs* TNF-α activates two receptors, TNF-R1 (P55TNF-R) and TNF-R2 (P75TNF-R) [61]. TNF-R1 and TNF-R2 have direct effects on neurons: TNF-R1 promotes neurodegeneration and causes neuronal death, while TNF-R2 promotes neuroprotection [76]. Activated TNFR1 induces ERK, JNK, p38 MAPK, NF-κB, and ceramide/sphingomyelinase pathways by TNF receptor-associated death domain (TRADD), TNF receptor-associated factor 2 (TRAF2), and receptor-interacting protein (RIP), which initiate prosurvival signaling, cellular proliferation, and cytokine production. TNF-ɑ can promote excitotoxicity by activation of glutamate-NMDA receptors and localization of α-amino-3-hydroxy-5-methyl-4-isoxazole propionic acid (AMPA) receptors to synapses [77].

*TGF-β and TGF-βR* In CNS, microglia, astrocytes and neurons express TGF-β1/2/3 and their receptors [78, 79]. TGF-β activates multiple intracellular pathways via its specific receptors and activates several MAPKs, including JNK, extracellular signal-regulated kinases (ERK1/2), PI3K, and p38-MAPK [80]. There is evidences that TGF-β increases in neurodegenerative diseases [79].

*Chemokines and chemokine receptors* During COVID-19, proinflammatory chemokines are produced in infected cells and can spread in general circulation and pass from the BBB. They then cause a neuroinflammatory response and stimulate their receptors on the surface of different CNS cell types. During neuroinflammation, chemokines have an important role in attracting inflammatory leukocyte subsets into the CNS [81]. CXCL10 exerts its critical role in controlling the entry of several important leukocyte subsets into the brain, where this chemokine is expressed by glia, neurons, and stromal cells in different CNS diseases. Initiation of the NF-κB pathway has been demonstrated in these cells [81]. Following exposure to viruses, astrocytes and microglia have a particular role in the expression of CXCL10 [81, 82]. The expression of CXCL10 can als be induced by IL-1β, TNF, and interferon (IFN) type I [81, 83]. CXCL12 is a secondary lymphoid chemokine, and its expression in neural cells is regulated by IL-1β [84]. The expression of CXCL12, SDF-1, and CXCR4 occurs in both glial and neuronal cells. CXCL12/SDF-1 axis induces proliferation of astrocytes. CXCR4 has been detected in all the analyzed areas of brain, including cortex, cerebellum, olfactory bulb, hypothalamus, hippocampus, striatum, and brain stem. ERK1/2 are two members of the MAPK family, and their activation is induced by CXCL12/SDF-1 in astrocytes [85]. The binding of CXCL12 to CXCR4 has significant roles in neuroinflammation [81]. Several studies have demonstrated that CCL2 levels were elevated during CNS inflammation and it is a critical neuroinflammatory mediator [86, 87]. In CNS, astrocytes and brain microvascular endothelial cells (BMEC) are the major source of CCL2 [86]. The CCL2/CCR2 axis activates the p38-MAPK signaling pathway [88].

### COVID-19–associated neurodegenerative diseases

The clinical occurrences of neurological manifestations associated with COVID-19, such as acute ischemic stroke (AIS), encephalopathy, acute necrotizing encephalitis, seizures, headaches, cerebral microbleeds, posterior reversible leukoencephalopathy syndrome, hemophagocytic lymphohistiocytosis (HLH), peripheral neuropathy, transverse myelitis, cranial nerve palsies, and demyelinating disorders, as well as neurodegenerative disease are rapidly increasing. An understanding of the neuroinflammatory mechanisms during COVID-19 in relation to the CNS may provide new insights into SARS-CoV-2 and neuroinflammation to better diagnose and workup neurodegenerative diseases.

### Alzheimer’s disease (AD)

Alzheimer’s disease (AD) is the most common dementing illness. In AD, release of inflammatory mediators triggers the innate immune response, which contributes to disease progression and severity [89]. Several mechanisms have been proposed to explain the potential roles of SARS-CoV-2 infection that could lead to the development of AD. A greater understanding of the common links between COVID-19 and AD would enable therapeutic strategies against both. AD and COVID-19 appear to have a mutualistic relationship. On the one hand, COVID-19 patients seem to be more susceptible to developing AD, while on the other hand, AD patients could be more vulnerable to severe COVID-19. Postmortem studies have revealed a significant increase in ACE-2 expression in the brain of AD patients [90]. In addition, genome-wide association studies (GWAS) revealed that the ACE-2 gene was expressed at a higher level in the brain tissue of patients with AD, with higher levels seen in patients with severe afflictions [91]. Recent research suggests ACE2 inhibitors can be used to treat neurodegenerative diseases, such as AD [92].

Recent studies have explained how the dysregulation of Eph/ephrin signaling is associated with Alzheimer’s disease, in particular, aberrant synaptic functions and cognitive impairment [93]. Elevation of caspase-1 activation can be observed in brains of patients with AD dementia, which caspase-1 needs for activation of pro-IL-1β and pro-IL18; thus suggesting a role for NLRP3 in AD [89, 94]. Activation of NLRP3 has been suggested as being crucial in the pathogenesis and treatment of severe COVID-19 infection [95]. Hence, these are some factors that make AD patients more prone to progressive neuropathies during COVID-19 infection, and by knowing these factors, new strategies for both can be devised during the COVID-19 pandemic. Interestingly, activation of the P2X7 receptor leads to the activation of inflammasome. P2X7 has been found to be associated with neurodegenerative diseases such as AD, PD, MS, and amyotrophic lateral sclerosis (ALS), and neuropsychiatric disorders such as depressive disorders, bipolar disorder, schizophrenia, and anxiety, as well as strokes and glioblastoma [16, 44–46]. In addition, COVID-19 and AD show common associations with proinflammatory markers such as IL-1, IL-6, cytoskeleton-associated protein 4 (CKAP4), galectin-9 (GAL-9 or Gal-9), and APOE4 allele [96]. Recent studies show that in peripheral blood of AD subjects, the levels of inflammatory cytokines increased, including TGF-β, TGF-α, IL-1β, IL-6, and IL-18 [97]. In AD, the major source of proinflammatory cytokines (IL-1β, IL-6, and TNF-α) are microglia and astrocytes [89]. Biomarkers of CNS injury in plasma and cerebrospinal fluid (CSF) have been reported in hospitalized patients suffering from mild to severe COVID-19, including neurofilament light chain protein, glial fibrillary acidic protein, S100b, astrocytic markers for injury, and total tau. However, further research is needed to confirm these biomarkers and identify new biomarkers in COVID-19-related neurodegenerative disorders. In addition, evaluating the existing studies and conducting new longitudinal cohort studies seem essential to monitor long-term outcomes of COVID-19 [98].

### Parkinson’s disease (PD)

Parkinson’s disease (PD) is the second most common neurodegenerative disease after Alzheimer’s disease (AD). It is characterized by the loss of dopaminergic neurons within the substantia nigra and the presence of α-synuclein aggregates (Lewy bodies) within the brain. The neurotropic properties of SARS-CoV-2 mean it may disrupt specific cellular processes that contribute to the pathogenesis of various neurological disorders, especially in PD and AD. In this regard, viral infection-induced colon inflammation, gut microbial imbalance, and upregulation of α-synuclein are of considerable interest considering the interaction between the gastrointestinal tract and the CNS (microbiome–gut–brain axis). Researchers have presented various viewpoints as different hypotheses discussed by neuroscientists about the association between SARS-CoV-2 infection and long-lasting neurodegenerative diseases, raising the question of whether COVID-19 may pose a risk factor for the development of neurodegenerative disorders such as PD. However, more investigations are needed to confirm these hypotheses [99]. Activated microglia were first observed by McGeer and colleagues in 1988 in the substantia nigra of patients with PD at postmortem [100]. In subsequent studies, an increase in the levels of proinflammatory cytokines, including IL-1β, IL-6, and TNF-α, were reported in the striatum of PD brains compared with healthy controls [101]. TNF-ɑ is one of the most important proinflammatory cytokines that is upregulated in PD [76]: high expression of NF-κB and TNF-α is related to HMGB1–TLR4 axis expression. On the other hand, mitophagy dysregulation is a critical factor in PD. Studies have demonstrated that Parkin-independent mitophagy and PINK1/Parkin-related PD led to mitochondrial dysfunction [102]. These pathways prevent mitochondrial antiviral signaling (MAVS) activation. By reducing downstream signaling pathways such as NF-κB and NLRP3 inflammasome, the production of proinflammatory cytokines is reduced [103]. A patient with PD can potentially increase the replication of the virus and help it escape the immune system [104]. COVID-19 patients had a significant rise in ephrin-A1 [38]: ephrin-A1 overexpression could lead to enhanced CXCL12 and CXCR4 binding. CXCL12/CXCR4 signaling pathways increase inflammation and neuropathology in the PD model in vivo and in vitro via ephrin-A1 [105]. Therefore, ephrin-A1 may be a good choice for decreasing the pathogenesis of PD in COVID-19 patients.

### Glioblastoma multiforma (GBM)

Glioblastoma multiforma (GBM), a common primary human brain cancer, is a highly infiltrative and devastatingly aggressive astrocytoma, which is characterized by resistance to apoptosis by chemotherapeutic treatment and radiation [106]. A vulnerable patient population may be GBM patients during the ongoing COVID-19 pandemic, mainly because of the high incidence of GBM and treatment-related immunosuppression, as well as the requirement for frequent hospitalizations [107]. In other words, COVID-19 infection and GBM have a common association with proinflammatory cytokines, chemokines, and Eph receptors. A recent study revealed that SARS-CoV-2 could directly infect some cells within human GBM tissues [108]. It was demonstrated that, in the brains of patients with GBM, the levels of proinflammatory cytokines, such as IL-1β and TNF-α, are elevated [106]. Moreover, 80% of anaplastic GBM tumors express TNF-α [63, 109]. In GBM, STAT3 is constitutively activated, and the levels of STAT3 and IL-6 secretion correlate with tumor grade in these patients [63, 110]. In cells that are infected by SARS-CoV-2 through ACE2, activation of STAT3 can occur [2, 111]. Furthermore, ephrin-B1 activation in astrocytes increased the level of STAT3 phosphorylation in vitro [35]. Thus, ACE2, as a specific receptor, and ephrin-B2, as a potential receptor for SARS-CoV-2, seem to have roles in STAT3 activation and are pathological factors in GBM during COVID-19. It has been demonstrated that pathological characteristics of GBM and COVID-19 have an association with CCL2, CCL5, and CXCL12 secretion [112, 113]. CXCL12 is overexpressed in GBM and activates its receptor CXCR4, causing an increase in the expression of CXCL10 and CCL2 [63, 114]. Utilizing the knowledge, we can adopt appropriate therapeutic strategies to reduce the effects of COVID-19 on GBM patients.

### Multiple sclerosis (MS)

Multiple sclerosis (MS) is a complex disease, generally considered to be an autoimmune disease, characterized by chronic inflammation, demyelination, and axonal pathology resulting in progressive neurological disabilities [115]. People with MS, compared with the general population, have an increased risk of infection by SARS-COV-2 [116]. IL-1β is a proinflammatory cytokine that exerts its role as an important component of MS pathogenesis and is detected in MS lesions [61, 117]. IL-1β regulates the expression of CXCL12 in neural cells. CXCL12 is a secondary lymphoid chemokine that has a pathologic role in MS and infiltrates leukocytes via enhancing the activation of CXCL12 receptor CXCR4 [84]. A high level of EphA1 may stimulate CXCL12 and CXCR4 binding [105]. CXCL10 also has a pathogenic role in MS [81], and TNF-ɑ is one of the most important proinflammatory cytokines, which is upregulated in MS [76]. IL-1β and TNF-α promote Th17, which produces IL-17, IL-21, and IL-22. IL-21 is one of the cytokines elevated in COVID19 [118]. It increases T cell subpopulations related to MS and plays pivotal roles in the severity and progression of the disease [119]. Furthermore, antigen presenting cells (APCs) are also detected in inflamed CNS tissue, which migrate to periventricular demyelinating lesions and cervical lymph nodes. They then present antigens to CD4^+^ T cells. In other words, APCs can kill glial cells and leave the axons exposed as a result of directly recognizing peptide epitopes on the surface of axons [120]. In COVID-19 patients, the Th17 cell number and the Th17/Treg ratio are elevated. Then, the expression of IL-17 and IL-23 is elevated [121]. A high level of IL-17 receptors are expressed by MS endothelial cells, which are permeable as a result of IL-17 stimulation as these cells play a key role in promoting inflammation of the CNS [120]. It may be possible to decrease the pathogenesis of MS by targeting EphA1.

### Neuro–Behçet’s disease (NBD)

Neuro–Behçet’s disease (NBD) is a neurological involvement in Behcet’s disease (BD), which is present in 5–30% of patients with BD, and causes devastating CNS damage [122]. SARS-CoV-2 activates inflammasomes, cytosolic protein complexes that activate caspase-1 or caspase-11 and cleave pro-IL-1β and pro-IL-18 cytokines to form their active forms [123]. One of the most relevant factor for autoinflammatory diseases such as BD are inflammasomes, which are characterized by IL-1β expression and innate immune system activation [123]. Therefore, the use of inflammasome inhibitors may be a promising therapeutic approach. A nonspecific inflammatory pattern compatible with autoinflammatory disease such as NBD is reflected by the release of proinflammatory cytokines including IL-1, IL-6, IL-8, IL-33, and TNF-α. Additionally, a number of studies have revealed an increase in the secretion of these cytokines during COVID-19 [122, 124]. In BD, anti-TNF-ɑ treatments such as infliximab and adalimumab are suggested in cases with a high level of resistance [125]. Furthermore, significant correlations have been observed between NF-κB and IL-33, and also among IL-33 and CCL2 (MCP-1) and CXCL10 (IP-10) in patients with NBD [122]. Therefore, we suggest that the administration of CXCL10 (IP-10) receptor CXCR3 antagonists and CCL2 (MCP-1) receptor antagonists, as well as treatments that reduce proinflammatory and inflammatory cytokines such as TNF-ɑ can be beneficial for patients with NBD infected with SARS-COV-2.

### Creutzfeldt–Jakob disease (CJD)

Creutzfeldt–Jakob disease (CJD), known as transmissible spongiform encephalopathy (TSE), causes rapidly progressive dementia, ataxia, pyramidal symptoms, and akinetic mutism [126]. Recently, in a case report of a patient with concurrent clinical presentation of COVID-19 and CJD, the authors hypothesized that systemic inflammatory mediators induced by COVID-19 might contribute to neurodegeneration and prion disease pathogenesis by triggering the loss of homeostatic identities in astrocytes and fostering neuroinflammatory transcriptional signatures. Furthermore, empirical evidence support this hypothesis in that there is inflammasome activation and increased secretion of the IL-1β, TNF-ɑ, and C1q cascades during COVID-19, as well as the fact that these factors are necessary and sufficient for activating A1 astrocytes that are involved in prion propagation [127].

### Encephalitis and hippocampal injury

MRI have revealed abnormal findings in cases with severe COVID-19, and a case report study showed abnormalities in the medial temporal lobe and hippocampus, which might result from encephalitis, hippocampal sclerosis, or post-convulsive encephalitis [128]. SARS-CoV-2 encephalitis has been reported in a few isolated cases, and SARS-CoV-2 has been detected in cerebrospinal fluid [113]. We suggest that SARS-CoV-2 may transmit from neuron to neuron from olfactory bulb to the hippocampus and influence this region through Ephs and ephrins as entry receptors, or trigger downstream signaling, leading to the production of proinflammatory cytokines and cell death. SARS-CoV-2 may target different areas of the hippocampus due to the expression of several Ephs and ephrins in these regions, which are linked to neurological symptoms such as transient global amnesia (TGA) [129]. Furthermore, it is possible to transmit SARS-CoV-2 from hippocampus to hypothalamus and influence this region as well, with the neurological symptoms, of this including vomiting [130] and appetite reduction [129].

### Ischemic stroke

Mao et al. observed six cases of acute cerebrovascular disease as neurological manifestations of COVID-19 [131]. In addition, in a tertiary center in New York, 19 cases of COVID-19-related stroke affecting the young and medium to large arteries were reported [132]. The World Stroke Organization (WSO) now recognizes that acute ischemic stroke (AIS) increases the severity of SARS-CoV2 viral infection 2.5 fold [133]. As well, there is a longstanding association between systemic infections, inflammation, and AIS [134]. There is also a possibility that hemorrhagic strokes might be caused by the cytokine storm, as described in a COVID-19 patient who developed acute necrotizing encephalopathy associated with late parenchymal brain hemorrhages [135].

Toll-like receptors (TLRs) may also have important roles in indirectly damaging neurons and causing ischemic stroke [23]. However, the exact role of intracellular TLRs, such as TLR3, TLR7, TLR8, and TLR9 in ischemic stroke remains unknown. There is a significant correlation between TLR7 and TLR8, and levels of proinflammatory cytokines such as IL-6, IL-1β, and TNF-α. Also, the activation of TLR7 and TLR8, together with other TLRs (but not TLR3 or TLR9) have a significant role in the pathophysiology of ischemic stroke through activation of the inflammatory response in CNS [136]. Therefore, blockade of TLR7 and TLR8 by TLR7 antagonists and TLR8 antagonists can be a potential treatment and protection from acute ischemic stroke in patients infected with SARS-CoV-2. It was also revealed that EphA2 receptors had an important role in ischemic stroke pathology. Taken together, early pharmacological targeting of proinflammatory cytokines (IL-1β, IL-6, and TNF-α) and their receptors, as well as EphA2 receptors following cerebral ischemia could be beneficial in decreasing tissue damage and neuronal loss in COVID-19 patients.

### Guillain–Barré syndrome (GBS)

Guillain–Barré syndrome (GBS) is characterized by an acute, generalized polyradiculoneuropathy, in which about two-thirds of cases manifest with asymptomatic infection, such as *Campylobacter jejuni*, Epstein-Barr virus, cytomegalovirus, or influenza. The association between GBS and COVID-19 has now been widely reported, though the strength and mechanism of the association, as well as the clinical and electrodiagnostic (EDx) patterns, remain unclear [137]. Respiratory infections are the most commonly reported symptoms of COVID-19 infection, and more than two-thirds of Guillain–Barré patients report respiratory infections before symptoms appear; therefore, the early cytokine storm in COVID-19 causes the release of several cytokines including IL-1β, IL-6, IL-17, TNF-α, and IFN-γ, as well as chemokines, which are mediators of the widespread and severe damage resulting in CNS and PNS damage, such as GBS. Several cytokines have been implicated in the pathogenesis of classic GBS, and it seems likely that the cytokine storm in COVID-19 may play a key role in the concurrent development of GBS and its rapid progression [138]. Taken together, GBS should be considered a neurological complication of COVID-19, and besides antiinflammatory and antiviral treatment, intravenous immunoglobulin (IVIG) or plasmapheresis therapy should be initiated for these patients.

### Future clinical perspectives

Various factors can be taken into consideration to investigate the potential prognostic and therapeutic strategies in the early stages of COVID-19 and neurodegenerative outcomes. The expression levels of ephrins and Eph receptors, NRP-1, P2X7, CD147, and TMPRSS2 in mouse and human brain cell lines during COVID-19 are suggested to clarify the possible fate of the COVID-19 toward neurodegenerative diseases. Therefore, in addition to ACE2, we can utilize a panel of these novel markers.

Administration of Eph receptor inhibitors may be a promising therapeutic approach in these patients. It is challenging for drug discovery to apply small molecules that can disrupt protein–protein interactions because the contact surfaces involved in these interactions are much larger (~1500–3000 A°) than those involved in protein–small-molecule interactions (~300–1000 A°). In spite of this, the ephrin-binding site of Eph receptors exhibits favorable characteristics for high-affinity binding of small molecules, allowing the discovery of a few classes of small molecules that bind EphA or EphB receptors [139]. Here are some examples: (a) derivatives of lithocholic acid (LCA), such as cholanic acid (ChA) and the L-Trp conjugate UniPR126, which are moderately selective for the EphA receptor subfamily [140]; (b) salicylic acid derivatives, which are selective inhibitors for EphA2 and EphA4 receptors [141]; (c) polyphenols found in green tea metabolites [142]; (d) doxazosin, an α1-adrenoceptor antagonist that has been recently shown to bind to EphA4 and EphA2 receptors [139, 143].

Moreover, drugs that block spike protein/CD147 interaction or CD147 expression can inhibit viral invasion and dissemination in progenitors/stem cells, as well as in CNS cells. Azithromycin is believed to reduce viral loads in COVID-19 hospitalized patients by interfering with ligand-CD147 receptor interactions. Apart from its effect on invasion, azithromycin decreases the expression of some metalloproteinases (downstream of CD147) and induces antiviral responses (interferons and interferon-stimulated genes) in primary human bronchial epithelial cells infected with rhinovirus, decreasing viral replication and release [144]. Doxycycline, a tetracycline analogue, reduced CD147 levels in a gallbladder carcinoma cell line [145] and gingival crevicular fluid (soluble form) in chronic periodontitis patients [146]. Additionally, humanized anti-CD147 antibodies such as meplazumab inhibit viral infection of cells by blocking CD147. According to studies, antibodies developed in preclinical research and clinical administration, such as meplazumab, metuximab, and metuzumab, all exhibit sound safety. Therefore, CD147 is a safe and reliable targeted drug. It is a receptor-blocking drug that inhibits viruses from invading host cells without being affected by virus variation [147]. It has also been suggested that anti-NRP-1 therapy can cause membrane NRP-1 shedding, resulting in elevated levels of soluble NRP-1 in circulation [148], which could further exacerbate side effects. Under cell culture conditions, antibodies targeting NRP-1 show a low inhibitory effect of viral infection [149]. Rather than systemic delivery (e.g.,, inhalable nanobodies that target NRP-1), delivering NRP-1-targeting therapies to key tissues may provide a better solution. There may be multiple mechanisms that assist virus entry into cells; therefore, targeting NRP-1 with peptides or monoclonal antibodies may not have significant beneficial therapeutic outcomes in clinical trials [150]. Regarding the role of P2X7 in neuroinflammation related to COVID-19, P2X7 receptor antagonism may be a novel strategy to prevent and treat neurodegenerative consequences [16]. Since all of these receptors can activate inflammasomes via NLRP3 activation, inflammasome inhibitors and IL-1β, IL-18, and IL-33 (because of shared signaling pathways) monoclonal antibodies or antagonists may be promising approaches for treating or avoiding neurodegenerative diseases.

A high level of cytokines is found in plasma from patients with severe COVID-19, especially IL-6, seen as a biomarker of inflammation and robust immune response. As a result of these efforts, clinical trials have been recruited for testing such a therapeutic strategy in hospitalized patients with COVID-19, using antibody anti-IL6 receptors (tocilizumab or sarilumab). In addition to its invasion inhibitory activity and antibiotic properties, azithromycin has immunomodulatory effects, which is why it has been prescribed to treat chronic inflammatory conditions such as bronchiolitis and rosacea. Although the underlying mechanisms of these antiinflammatory effects are still unclear, some studies have shown that azithromycin treatment reduces IL-6 levels [144, 151]. The launch of clinical trials with virus-neutralizing antibodies targeting SARS-CoV-2 may be promising in eliminating the virus, preventing infection, and reducing the viral load [150], which paves the way toward virus-side treatments rather than host-side targeting. Interestingly, ACE2, IL-6R, and EphA/B transduce through the JaK2/STAT3 signaling pathway; therefore, pan JaK inhibitors (JaKinibs) such as baricitinib, tofacitinib, etc. may be promising for concomitantly targeting several discrete signaling pathways in a neuroinflammation context [3].

## Conclusion

The COVID-19 pandemic has already influenced the routine process of diagnosing and treating patients with neurodegenerative disease. Investigating the potential receptors for SARS-CoV-2 entry to host cells, the cytokine storm, and neuroinflammation induced by the immune system may provide a comprehensive and novel insight into the pathological effects of COVID-19 on the central nervous system and the development, acceleration, or deterioration of neurodegenerative diseases. Thus, understanding these markers and their mechanisms of action may lead to new diagnostic and therapeutic approaches to adopt appropriate and more timely policies to inhibit or minimize long-lasting pathological neuroinflammatory outcomes of COVID-19. In this regard, we have mechanistically unraveled potential receptors for SARS-CoV-2 entry to the CNS, including ACE2, NRP-1, CD147, P2X7, ephrin ligands and Eph receptors, and cytokines that may result in various neurodegenerative disorders associated with COVID-19, and suggest targeting them as promising therapeutic approaches. Moreover, inflammasome inhibitors and JaKinibs activated downstream of these receptors may be novel strategies to prevent or treat neurodegenerative diseases.

## Data Availability

Not applicable.

## Abbreviations

AD: Alzheimer’s disease
BBB: Blood–brain barrier
COVID-19: Coronavirus disease 2019 (COVID-19)
CNS: Central nervous system
Ephrins: Eph family receptor-interacting proteins
GBS: Guillain–Barré syndrome
MS: Multiple sclerosis
NLRP3: Nod-like receptor family pyrin domain-containing 3
NRP-1: Neuropilin-1
PD: Parkinson’s disease
PNS: Peripheral nervous system
SARS-CoV-2: Severe acute respiratory syndrome coronavirus 2
TMPRSS2: Transmembrane serine protease 2
